# Enhancement of lower limb motor imagery ability *via* dual-level multimodal stimulation and sparse spatial pattern decoding method

**DOI:** 10.3389/fnhum.2022.975410

**Published:** 2022-08-11

**Authors:** Yao Hou, Zhenghui Gu, Zhu Liang Yu, Xiaofeng Xie, Rongnian Tang, Jinghan Xu, Feifei Qi

**Affiliations:** ^1^Mechanical and Electrical Engineering College, Hainan University, Haikou, China; ^2^College of Automation Science and Engineering, South China University of Technology, Guangzhou, China; ^3^School of Internet Finance and Information Engineering, Guangdong University of Finance, Guangzhou, China; ^4^Pazhou Lab, Guangzhou, China

**Keywords:** brain-computer interface, motor imagery, stimulation, group lasso, common spatial pattern

## Abstract

Recently, motor imagery brain-computer interfaces (MI-BCIs) with stimulation systems have been developed in the field of motor function assistance and rehabilitation engineering. An efficient stimulation paradigm and Electroencephalogram (EEG) decoding method have been designed to enhance the performance of MI-BCI systems. Therefore, in this study, a multimodal dual-level stimulation paradigm is designed for lower-limb rehabilitation training, whereby visual and auditory stimulations act on the sensory organ while proprioceptive and functional electrical stimulations are provided to the lower limb. In addition, upper triangle filter bank sparse spatial pattern (UTFB-SSP) is proposed to automatically select the optimal frequency sub-bands related to desynchronization rhythm during enhanced imaginary movement to improve the decoding performance. The effectiveness of the proposed MI-BCI system is demonstrated on an the in-house experimental dataset and the BCI competition IV IIa dataset. The experimental results show that the proposed system can effectively enhance the MI performance by inducing the α, β and γ rhythms in lower-limb movement imagery tasks.

## 1. Introduction

Brain-computer interfaces (BCIs) allow people to communicate or control external devices directly by using information from the brain without relying on the peripheral nervous system and muscles (Bulárka and Gontean, 2016; Abiri et al., 2019; Jin et al., 2021; Sun et al., 2021). The BCI technology has exhibited tremendous potential in motor function assistance and rehabilitation engineering for disabled or stroke patients (Bai et al., 2020; Mane et al., 2020; Miao et al., 2020). In particular, motor imagery (MI) BCI systems with stimulation, referred as enhanced MI-BCI, are receiving increasing attention in the rehabilitation field because MI-BCI systems with stimulation can provide a non-invasive and active way to analyze the relationship between the limbs stimulation and brain activities (Hwang et al., 2009; Herrador Colmenero et al., 2018; Vourvopoulos et al., 2019). In the development of the enhanced MI-BCI system, two major challenges are the paradigm design of the stimulation and EEG decoding methods.

The performance of a enhanced MI-BCI system is highly dependent on the stimulation paradigm design. Many external stimuli, such as visual, auditory, proprioceptive, and functional electrical stimulation (FES), can provide stimulation from external devices to the human body and cortex. The remaining problem is how to design a stimulation paradigm for disabled or stroke patients to improve the performance of rehabilitation training (Foong et al., 2019). In recent years, various stimulation methods have been proposed for motor imagery rehabilitation training. For instance, in Boord et al. (2010), lower limb movement imagery BCI with visual stimulation was proposed to strengthen the desynchronization rhythm during imaginary movement by watching a video of the legs being raised in lower limb movement imagery. In McCreadie et al. (2014), an MI-BCI with auditory stimulation was proposed to explore the difference in feedback between visual and auditory stimuli, suggesting that auditory stimulation is the equivalent substitute of visual stimulation. In Chatterjee et al. (2007), an MI-BCI with tactile stimulation was designed to help subjects regulate contralateral imaginary tasks. In Do et al. (2012), a closed-loop BCI with FES stimulation was used to improve foot function in stroke survivors. FES stimulation can produce a more accurate and stronger neural function activation effect in the motor cortex under the action of patients' subjective motor imagery. Furthermore, MI-BCI systems with multimodal stimulation have also been applied for MI training. In Wang et al. (2019), a BCI game with visual stimulation and auditory stimulation was designed to improve patients' attention focus during lower-limb rehabilitation training. In Vukelić and Gharabaghi (2015), a closed-loop BCI with stimulation from visual, haptic, and proprioceptive modalities was designed to strengthen the activation characteristics of the sensorimotor cortex of the brain. In Ono et al. (2018), an MI-BCI system with visual and proprioceptive stimulation was proposed to enhance motor imagery ability. This shows that the motor imagery event-related desynchronization (MI-ERD) power of the subjects accepting multimodal stimulation, significantly increased after training. However, the above stimulation paradigms mainly focus on improving the ability of motor imagery and performance of enhance MI-BCIs, few of them considering the combination of multimodal stimulation and effect of stimulation on α, β, and γ rhythms, respectively.

On the other hand, the effective decoding of EEG signal is also very important for the enhanced MI-BCI system performance. Common space pattern (CSP) is one of the most widely used methods for feature extraction in motor imagery EEG signal decoding (Blankertz et al., 2007; Barachant et al., 2010). CSP is designed to learn a spatial filter to extract low-dimensional features by maximizing the variance of one class while minimizing the variance of the others. Many extensions of CSP have been proposed to optimize and improve the performance of CSP. For example, the filter bank CSP (FBCSP) was designed to learn spatial filters from multiple frequency sub-bands and to select the most significant features based on mutual information (Ang et al., 2008). A regularized CSP (RCSP) was proposed to obtain more discriminative features by considering the prior information of the covariance matrices as regularization criteria (Lotte and Guan, 2010). A sparse CSP (sCSP) method was designed to learn sparse filters by a greedy search based generalized eigenvalue decomposition approach and subset of channels contributes to feature extraction (Goksu et al., 2011). The correlation-based channel selection RCSP was designed to select the channels containing the most relevant information based on Pearson's correlation coefficient and efficient spatial features were extracted using regularized general spatial patterns (Jin et al., 2019). The Riemannian CSP was proposed to learn spatial features by replacing the Euclidean mean in the original CSP with the Riemannian mean (Barachant et al., 2010). Recently, deep learning methods with strong fitting ability for mass data have been developed for the rapid decoding of EEG signals using MI-BCI systems (Lawhern et al., 2018). Graph convolutional neural networks (GCNs-Net) was designed to filter EEG signals based on functional topological relationship to learn generalized features using graph convolutional layers (Lun et al., 2020). Scout EEG source imaging (ESI) with convolutional neural network (CNN) was proposed to solve the EEG forward and inverse problems using the technique of scout EEG source imaging (ESI) to extract features from the time series of scouts based on the Morlet wavelet approach (Hou et al., 2020). Both CSP-based methods and deep learning methods can learn efficient features from EEG signals. However, the enhanced MI-BCI system, employing a few of the above methods, can not correctly identify the frequency sub-bands relevant to the stimulation, which can result in a biased analysis of stimulation effects.

**Figure 1:**
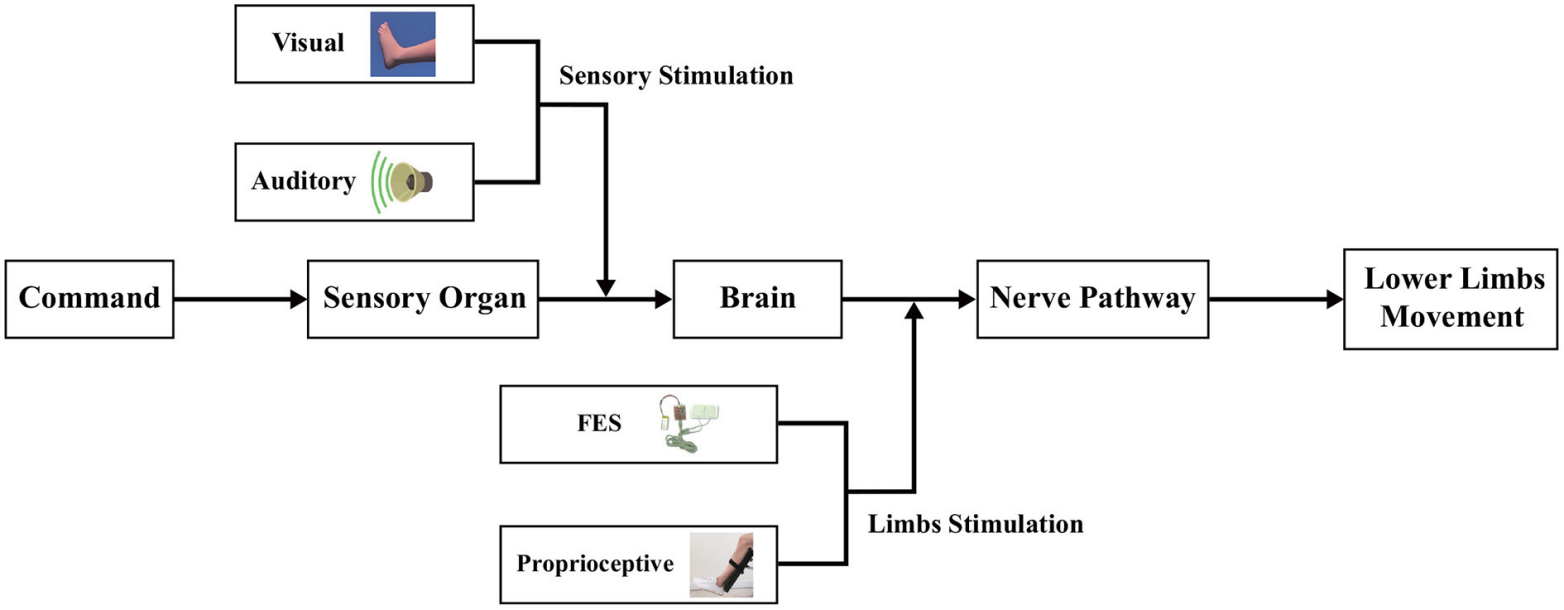
The framework of the multi-modal dual-level stimulation paradigm. The visual and auditory stimulations act on the sensory organ while the FES and tactile stimulations act on the lower limb.

Although many stimulation paradigms and decoding methods in MI-BCI systems have been proposed to improve the performance of motor function rehabilitation, they are mainly designed for single-modal stimulation or multimodal mixed stimulations, and only a few of these methods can select the best frequency sub-bands related to stimulation to classify the EEG signal. In view of the shortcomings of the above two challenges, in this study, a multimodal dual-level lower-limb MI-BCI system is proposed for rehabilitation training, and an upper triangle filter bank sparse spatial pattern (UTFB-SSP) is designed to select the optimal frequency sub-bands and improve the decoding performance. As shown in Figure 1, the multimodal dual-level stimulation paradigm consists of the four types of stimulation: visual, auditory, FES, and proprioceptive. Notably, the visual and auditory stimulations directly act on the sensory organ (sensory stimulation), while FES and proprioceptive stimulations act on the lower limb (limbs stimulation). In addition, to achieve high decoding performance, the proposed UTFB-SSP method utilizes the group lasso to automatically select the optimal frequency sub-bands from the upper triangle filter bank. Recently, similar MI-BCI systems with multimodal stimulation have also been proposed for lower-limb rehabilitation training (Ren et al., 2020). In such systems, the FES was adopted as an additional enhancement mode to improve the subject's attention on the associated lower limb, while the virtual reality (VR) was designed to provide visual guidance on enhancing imagery abilities. FBCSP was applied to decode the EEG signal. The main difference between the proposed method and other similar methods is that the proposed system can provide a complex and efficient stimulation paradigm in the form of a dual-level instead of an overlap-level form. The contributions of this study are as follows:

- A multimodal stimulation paradigm was designed to enhance the MI performance. Visual, auditory, FES, and proprioceptive stimuli were performed on the sensory organ and lower limb, respectively.
- A novel UTFB-SSP method was proposed for the analysis of EEG signals. The UTFB-SSP can identify the optimal frequency sub-bands related to stimulation as well as analyze the effects of different stimulations.
- After combining the stimulation paradigm and decoding method, a multimodal dual-level lower-limb MI-BCI system was designed to reveal the relation between brain activity and lower-limb movements.

The remainder of this paper is organized as follows. In Section 2, the experimental setup and EEG decoding method are described. In Section 3, an extensive experimental analysis is presented to demonstrate the effectiveness of the proposed system. Finally, some conclusions are presented in Section 4.

## 2. Materials and methods

### 2.1. Participants

Ten able-bodied male volunteers (aged 22 ± 3) participated in the study. All of the participants provided written informed consent.

### 2.2. Apparatus and instrumentation

**Figure 2:**
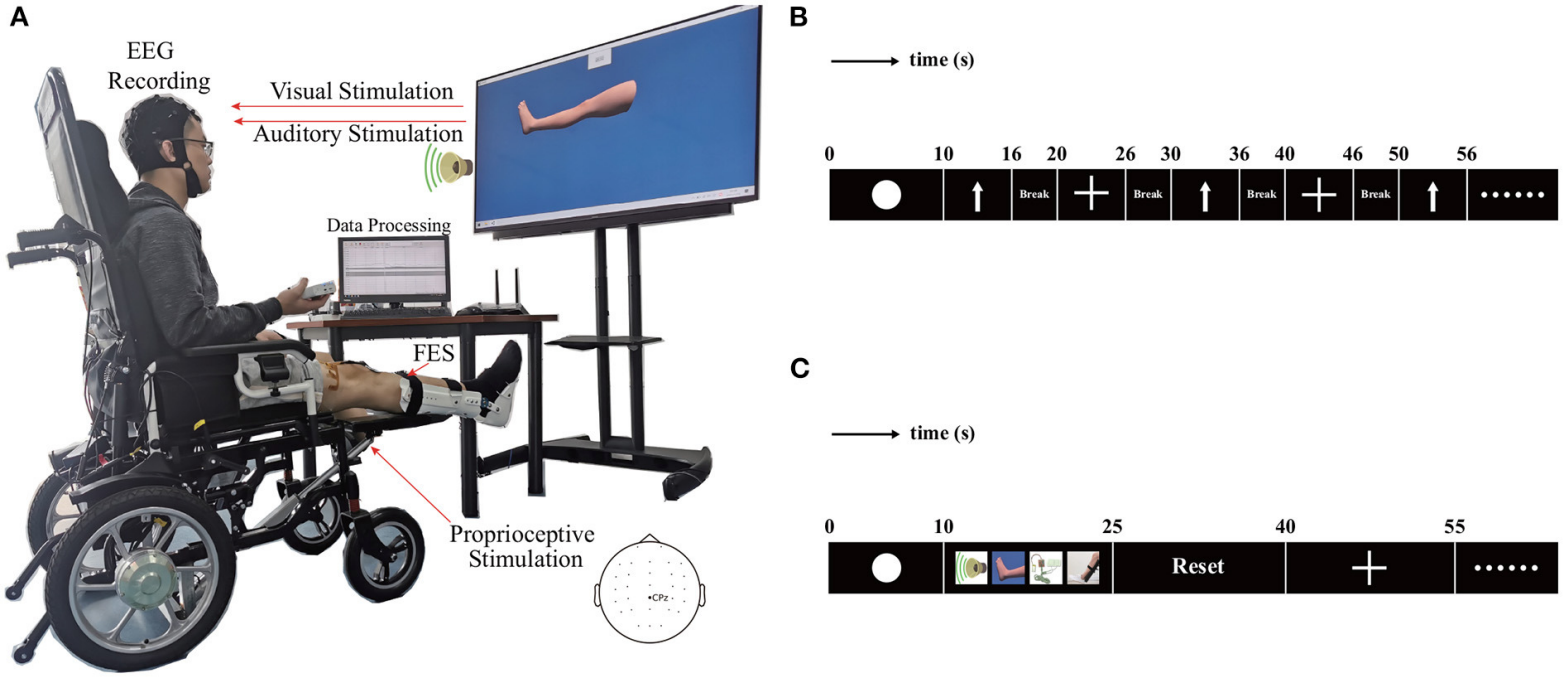
The hardware structure and experimental protocol for the proposed BCI system. **(A)** Hardware structure. **(B)** Paradigm of experiment 1. **(C)** Paradigm of experiment 2.

As shown in Figure 2A, the hardware structure of the multimodal dual-level lower-limb MI-BCI system mainly includes an EEG signal recording subsystem, dual-level stimulation subsystem, and data processing subsystem.

#### 2.2.1. EEG recording

A 30-channel active Bio-Signal data acquisition system (Poseidon, China) was used to acquire the EEG signals. The electrodes were placed on the scalp at locations overlying the motor cortices. The Pz electrode was used for positioning according to the specifications of the International 10-20 Electrode System. The ground electrode was placed on AFz and the reference electrode was placed on the left earlobe. All impedances were maintained below 20 kΩ at the onset of each session. The sampling rate was set to 250 Hz.

#### 2.2.2. Audiovisual stimulation

The visual stimulation consisted of a video of the lower limbs laid down or being raised while the participant performed the lower limb movement imagery. Auditory stimulation refers to the notification sound of “Please raise your legs.” In a motor imagery task, visual and auditory stimulations were simultaneously played to notify the participant. The human brain can directly receive audiovisual stimulation to enhance rhythm desynchronization during motor imagery, which is referred to as sensory stimulation in this paper.

#### 2.2.3. Proprioceptive stimulation and FES

Proprioceptive stimulation is a single-of-freedom mechatronic device installed on a wheelchair. During the motor imagery task, the participant sitting in a wheelchair places his right leg on electric pedals. When mechatronic device receives computer commands, it can actively raise the participant's right leg using an electric lift pedal. The FES includes two pairs of self-adhesive surface electrodes fitted on the right leg of the participant. The frequency of electrical stimulation was set to 5 Hz, and the voltage amplitude was set to 5 V. In the motor imagery task, the proprioceptive and electrical stimulations were simultaneously performed on the participant's right legs. They are referred to as limbs stimulation.

### 2.3. Experimental protocol

To evaluate the performance of multimodal stimulation, two experiments were designed for a fair comparison. The lower limb motor imagery experiment without stimulation was regarded as the control group. The experiment of lower-limb motor imagery with multimodal stimulation is an experimental group.

#### 2.3.1. Experiment I: Lower limb motor imagery without stimulation

The procedure of each trial is shown in Figure 2B. During the first interval (0–10 s), the screen kept blank. The signal from the initial 10 s was taken as the baseline. Next, an arrow cue pointing up or a cross cue presented and kept on the screen for 10–16 s. The cross represented the resting state. The participant was required to begin imagining the movement of the leg immediately after the arrow appeared, and participants were asked to continue with MI until the arrow disappeared from the screen. The participants rested for a period of 4 s between the trials. During the trials, they were presented with cues in random order in blocks of 50 trials, with each cue comprising 25 trials. Each participant completed four blocks, for a total of 100 trials for the arrow and 100 trials for the cross. A single block lasted for approximately 9 min, and the participant was given the opportunity to have a 5-min break before proceeding to the next block of trials. This break allowed the participant to relax and minimize the potential for fatigue.

#### 2.3.2. Experiment II: Lower limb motor imagery with multi-modal stimulation

The structure of each trial is illustrated in Figure 2C. During the initial interval (0–10 s), the screen remained blank. The signal from the first 10 s constituted the baseline. Four types of stimulation were provided during motor imagery. This stimulation included a video of the leg lift, voice prompt, FES and pedal lifting the leg. The video of the leg lift appeared and remained on the screen from 10 to 25 s. The participants were required to imagine imitating the movement displayed in the video. The voice prompt was played from 10 to 25 s. The participant was fitted with two pairs of self-adhesive surface electrodes on the right lower leg. The FES system provided electrical stimulation from 10 to 25 s. The electric lift pedal lifted the right leg from 10 to 25 s. At the end of motor imagery, the pedal returned to the starting position from 25 to 40 s. Next, a cross cue appeared and remained on the screen from 40 to 55 s to allow the participant to rest. The blocks of 40 trials comprised 20 trials for each cue. Each participant completed five blocks, for a total of 100 trials for the arrow and 100 trials for cross. A single block lasted for about 15 min, and the participant was given the opportunity to have a 5-min break before proceeding to the next block of trials.

### 2.4. EEG processing algorithm

**Figure 3:**
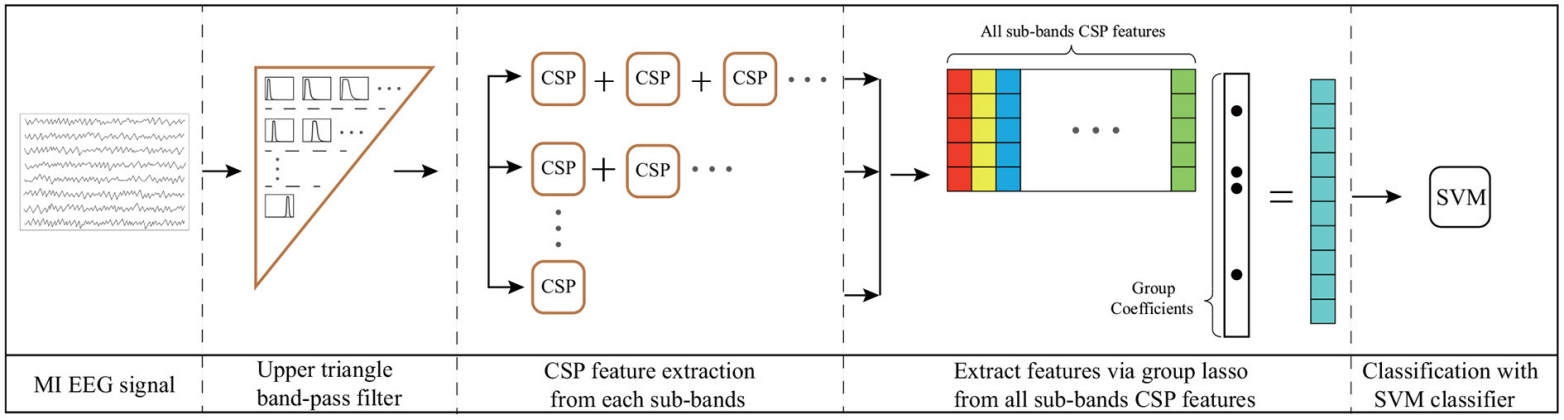
Architecture of UTFB-SSP algorithm. 1) Upper triangle band-pass filter; 2) CSP feature extraction from each sub-band; 3) Extraction of features *via* group lasso from all sub-bands of CSP features; 4) Classification *via* SVM classifier.

In this section, a UTFB-SSP is proposed for the decoding of EEG signals. The UTFB-SSP algorithm is illustrated in Figure 3. The UTFB-SSP method comprises the following four progressive stages of feature extraction and classification for an EEG signal: upper triangle filter bank construction, spatial feature extraction from each sub-band, feature selection using the group lasso (GL) (Yuan and Lin, 2006) method, and classification *via* the support vector machine (SVM) classifier.

First, a large frequency band from 4 to 42 Hz is decomposed into 90 sub-bands, which are arranged in an upper triangle form, that is,


(1) $$\begin{array}{l} \left[4-8Hz,4-12Hz,...,\;...,\;...,4-42Hz\right]\\ \left[6-10Hz,6-14Hz,...,\;...,6-42Hz\right]\\ \left[8-12Hz,8-14Hz,\;...,8-42Hz\right]\\ ......\\ {}[38-42Hz]. \end{array}$$

The proposed upper triangle filter bank (UTFB) extracts multiple overlapping sub-bands capturing abundant information on θ (4–8 Hz), α (8–13 Hz), β (14–30 Hz), and γ (30–42 Hz) rhythms. Denoting an *N*-channel EEG signal recorded from an MI-based BCI system as **X**(*t*) ∈ ℝ^*N*×*L*^, the **X**(*t*) are subsequently bandpass-filtered into 90 subfrequency bands.

For **X**^*i*^(*t*) corresponding to the *i*-th sub-band, CSP was applied to learn the spatial features. The spatial filter matrix ${\text{W}}^{i}\in {\mathbb{R}}^{N\times N_{s}}$ of CSP can be learned by maximizing the variance of the lower-limb movement trials while minimizing the variance of resting trials


(2) $$\begin{array}{r} \begin{array}{c} Max/Min\;\;J(W^{i})=\frac{\text{tr}({\text{W}}^{i^{T}}{\text{C}}_{1}{\text{W}}^{i})}{\text{tr}({\text{W}}^{{}^{i^{T}}}{\text{C}}_{2}{\text{W}}^{i})} \end{array} \end{array}$$

where **C**_*j*_ is the arithmetic mean of the covariance matrices of **X**^*i*^(*t*) belonging to class *j*. The optimal matrix **W**^*i*^ is composed of the eigenvectors of ${\text{C}}_{2}^{-1}{\text{C}}_{1}$, that correspond to the first $\frac{N_{s}}{2}$ largest and $\frac{N_{s}}{2}$ smallest eigenvalues. Letting **z**^*i*^ denote the CSP features corresponding to the *i*-th sub-band


(3) $$\begin{array}{r} {\text{z}}^{i}=\mathrm{diag}\left({{\text{W}}^{i}}^{T}{\text{X}}^{i}{{\text{X}}^{i}}^{T}{\text{W}}^{i}\right) \end{array}$$

The CSP features from all sub-bands are then merged into a high-dimensional vector, **u** = [**z**^1^, **z**^2^, …, **z**^90^].

Finally, the group lasso was applied to select the crucial group of CSP features from the high-dimensional vector **u**. The group lasso is an extension of the lasso to do variable selection on (predefined) groups of variables in linear regression models. It selects the entire group elements together and a lasso penalty function is applied to the L2-norm of the coefficients within each group. It force a whole set of coefficients to become zero, in other words, to eliminate a whole set of variables. It has the attractive property that it does variable selection at the group level and is invariant under (groupwise) orthogonal transformations like ridge regression. The loss function of the group lasso is expressed as follows:


(4) $$\begin{array}{r} \underset{\text{a}}{\min}\overset{m}{\underset{i=1}{\sum }}{\left(y_{i}-{\text{a}}^{T}{\text{u}}_{i}\right)}^{2}+\text{λ}\overset{k}{\underset{i=1}{\sum }}||{\text{a}}_{Gi}|{|}_{2} \end{array}$$

where *m* and *k* denote the number of trials and groups, respectively. The first term of (4) controls the fit of the model to the data, while the second term controls sparsity of the group. The coefficient of **a** represents the weight of the features of all trials, and the coefficient of **a**_*Gi*_ represents the weights of the *i*-th group features. The parameter λ is used as the group-wise regularization penalty. After selecting the crucial group features **u**_*s*_ by group lasso, the SVM classifier is applied for classification. The pseudocode for the UTFB-SSP is provided in Algorithm 1.

**Algorithm 1:** Upper triangle filter bank sparse spatial pattern (UTFB-SSP).

**Input:** Given training datasets *X*_*tr*_ with known classes;
**Input:** Given test data *X*_*te*_ with unknown classes;
**Output:** Label *y* of test data *X*_*te*_;
1: Decompose a given frequency band into multi sub-bands based on the upper triangle form;
2: Apply Butterworth band-pass filter to each sub-band of training and test data;
3: Use Equation (3) to obtain spatial features from training and test data;
4: Merge all CSP features into a high-dimensional vector **u**;
5: Use Equation (4) to select crucial features **u**_*s*_ from **u**;
6: Identify the selected features of test data using the SVM classifier;

## 3. Results and discussion

In this section, initially, the data and competing methods are briefly reviewed. Subsequently, the effectiveness of the dual-level stimulation paradigm and the performance of the proposed UTFB-SSP algorithm are evaluated. Finally, an extensive discussion is provided on enhanced MI-BCI systems.

### 3.1. Data and algorithm description

Two datasets were used to demonstrate the effectiveness of the proposed method: an in-house dataset collected from the experimental protocol in Section 2 and a public dataset from BCI competition IV.

1) An in-house dataset was recorded from ten subjects (A01–A10) who performed experiment1 and experiment2. The recorded signals consisted of 30 EEG channels. For each subject, there were two types of EEG for the lower limb motor imagery and rest conditions, with 70 training and 30 test trials for each EEG condition. Thus, the overall number of training/test trials for each subject was 140/60.
2) Dataset IIa of BCI competition IV was recorded from nine subjects (S01–S09) who performed foot motor imagery tasks and breaks. The recorded signals consisted of 22 EEG channels. There were 72 training and 72 test trials for each subject and mental task. Thus, the overall number of training and test trials for each subject was 144/144.

To evaluate the performance, four competing algorithms for EEG decoding were used as follows:

1) CSP+SVM: CSP followed by SVM classifier (Barachant et al., 2010).
2) FBCSP+SVM: FBCSP decomposes the EEG into multiple sub-bands, extracting the CSP features at each sub-band, and selecting features based on mutual information, with SVM performing the classification (Ang et al., 2008).
3) ESI+CNN: This approach combines the scout EEG source imaging (ESI) technique with a convolutional neural network (CNN) for the classification of motor imagery (MI) tasks (Hou et al., 2020).
4) GCNs-Net: A graph convolutional neural network for decoding motor imagery signals (Lun et al., 2020).
5) EEGNet: A compact convolutional neural network for EEG-based BCIs (Lawhern et al., 2018).

In this study, parameter was determined by cross-validation. For each subject, the number of CSP spatial filter was set as {11, 6, 6, 4, 10, 2, 6, 2, 2, 3} for in-house dataset (without stimulation), {3, 8, 5, 13, 4, 3, 6, 2, 2, 6} for in-house dataset (without stimulation), and {3, 2, 2, 6, 2, 3, 2, 2 ,6} for BCI competition IV dataset. The group-wise regularization penalty λ was set as {0.8, 0.15, 0.5, 0.15, 0.4, 0.2, 0.75, 0.05, 0.5, 0.2} for in-house dataset (with stimulation), {0.2, 0.05, 0.05, 0.55, 0.05, 0.05, 0.2, 0.05, 0.9, 0.75} for in-house dataset (with stimulation), and {0.55, 0.2, 0.3, 0.15, 0.1, 0.7, 0.45, 0.05, 0.85} for BCI competition IV dataset.

### 3.2. Effectiveness of dual-level stimulation paradigm

**Figure 4:**
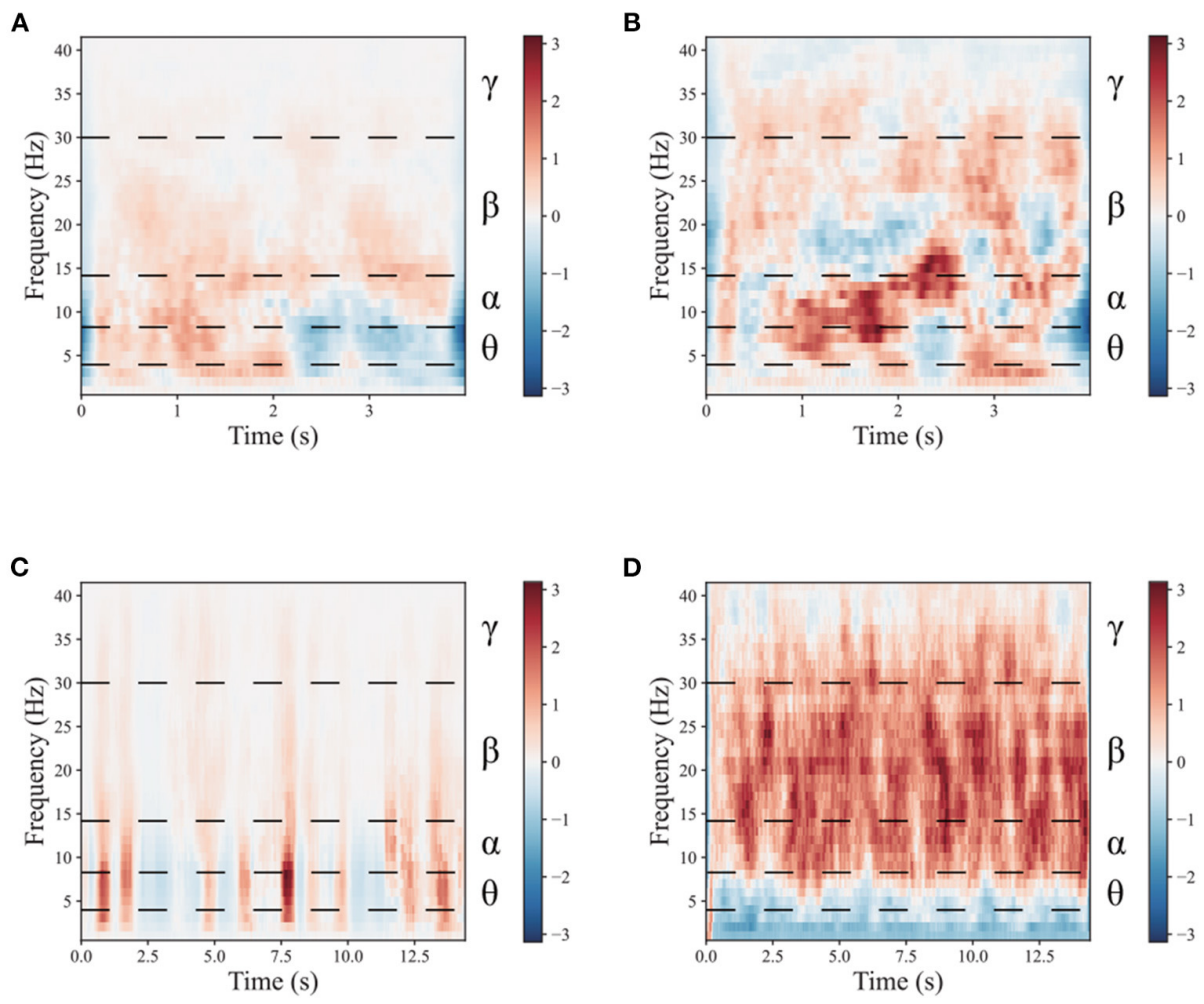
The mean time-frequency of the lower limb movement imagery with and without stimulation in CPZ electrodes from subject A09 of the in-house dataset. **(A)** The rest state imagery without stimulation. **(B)** Lower limb movement imagery without stimulation. **(C)** The rest state imagery with stimulation. **(D)** Lower limb movement imagery with stimulation.

**Figure 5:**
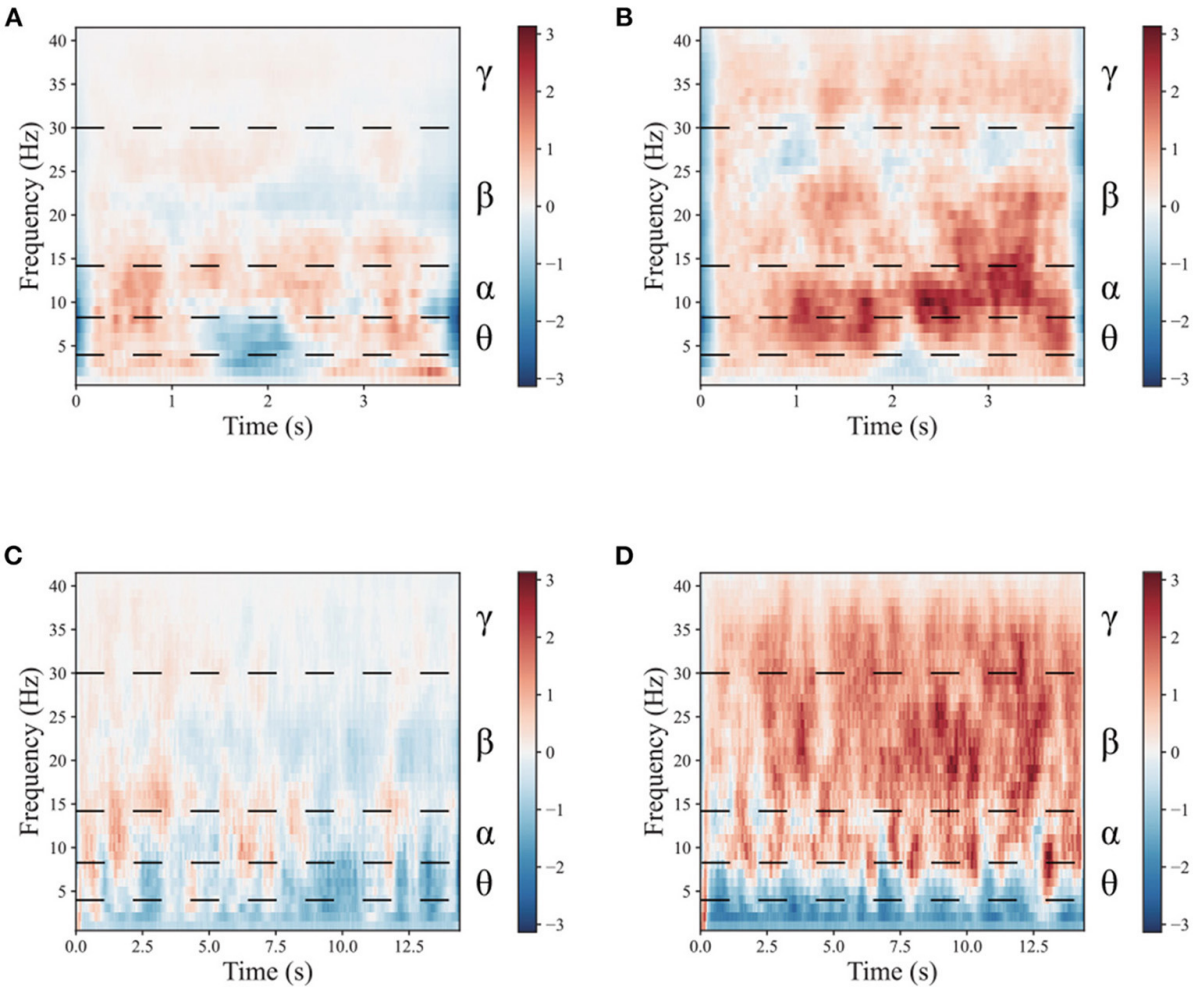
The mean time-frequency of the lower limb movement imagery with and without stimulation in CPZ electrodes from all subjects of the in-house dataset. **(A)** The rest state imagery without stimulation. **(B)** Lower limb movement imagery without stimulation. **(C)** The rest state imagery with stimulation. **(D)** Lower limb movement imagery with stimulation.

To demonstrate the benefits of the multimodal dual-level stimulation paradigm as a powerful tool for enhancing MI performance, a comparison is provided to analyze the effects of the proposed stimulation paradigm. Figures 4, 5 shows the time-frequency diagram of lower limb movement imagery with and without stimulation. θ waves lie within the range of 4–8 Hz, α with a rate of change lies between 8 and 13 Hz, β, the rate of change lies between 13 and 30 Hz. β is the brain wave usually associated with active thinking, active attention, focus on the outside world or solving concrete problems and finally the γ waves which lie within the range of 30 Hz and up. It is thought that this band reflects the mechanism of consciousness. In this study, the filtering range cover θ, α, β, and γ rhythms. These rhythms are the best frequency bands for motor imagery. And They are marked in time-frequency diagrams. In the comparison of (a,b), it is observed that the α rhythm appears in the lower limb movement imagery without stimulation. In (c,d), it is observed that the α, β, and γ rhythms appear simultaneously in the lower limb movement imagery with multimodal stimulation. Combining the results of (a–d), the designed paradigm with visual, auditory, FES, and proprioceptive stimulations could greatly enhance the α, β, and γ rhythms to help the brain better understand lower limb movement imagery.

**Figure 6:**
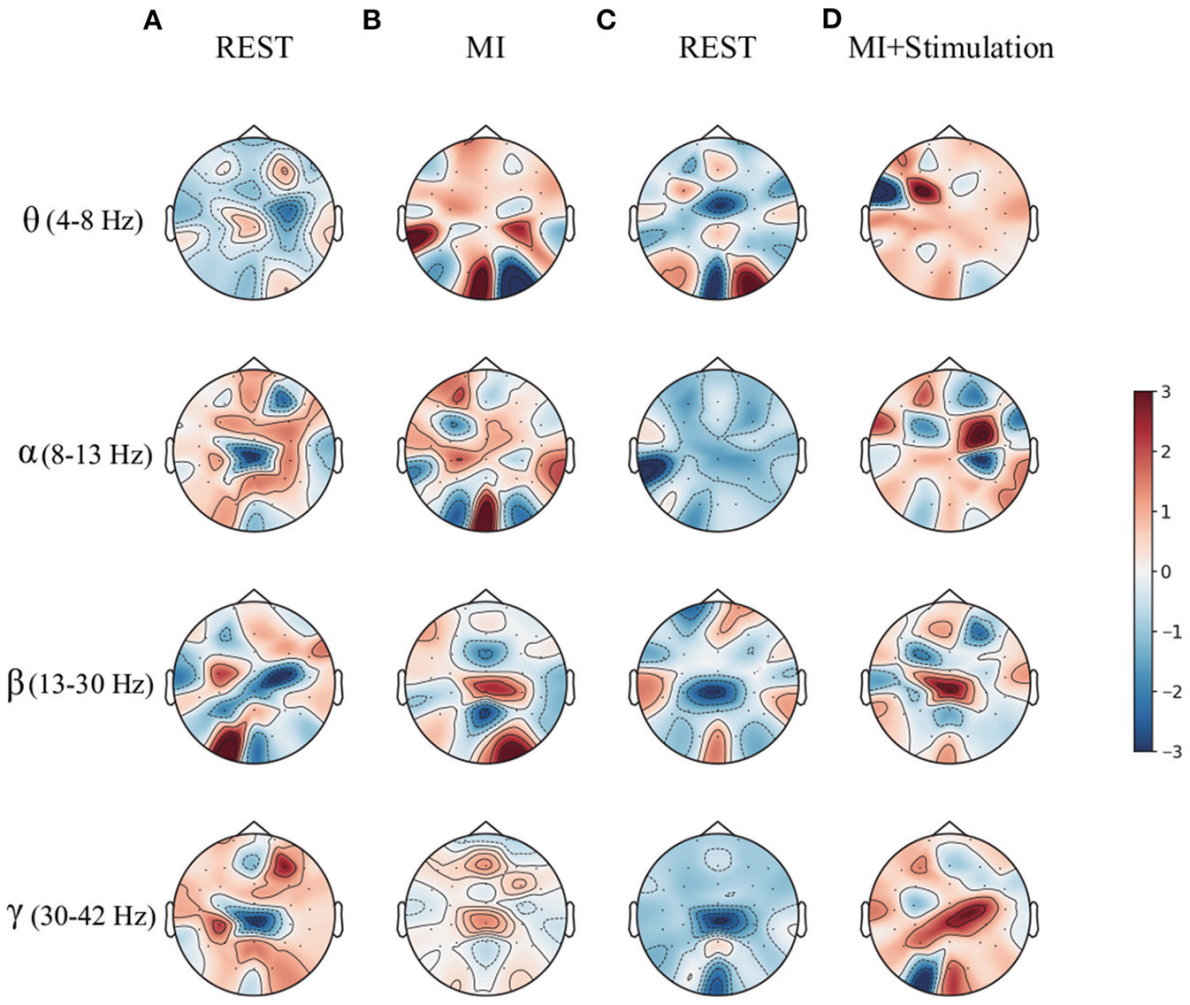
The topographic map of the spatial filter for the lower limb movement imagery with and without stimulation from subject A09 of in-house dataset. **(A)** The rest state imagery without stimulation. **(B)** Lower limb movement imagery without stimulation. **(C)** The rest state imagery with stimulation. **(D)** Lower limb movement imagery with stimulation.

**Figure 7:**
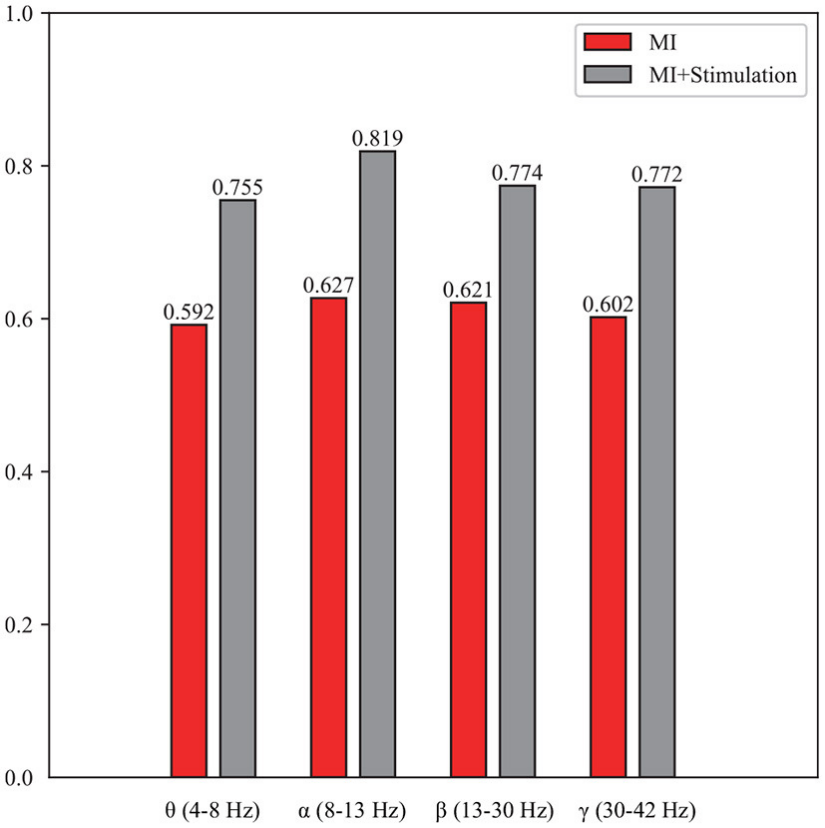
The classification performance of the lower limb movement imagery with and without stimulation for θ, α, β, and γ rhythms.

To enrich the comparison, a topographic map of the spatial filter was also presented for lower-limb movement imagery with and without stimulation in Figure 6. Compared with the spatial filter learned from pure motor imagery in Figures 6A,B, it is observed that the spatial filter from the stimulation (Figures 6C,D) has a larger difference in the surrounding CPZ electrode area, which is dedicated to the lower limb imagery movement. More specifically, the classification performance of lower limb movement imagery with and without stimulation in θ, α, β, and γ rhythms, is further demonstrated. Figure 7 shows the mean accuracy of the A01–A10 subjects on the in-house dataset with respect to the four rhythms. As shown in Figure 7, the accuracies on movement imagery with stimulation are significantly higher than the accuracies on movement imagery without stimulation. These results reveal that the proposed multimodal stimulation paradigm can effectively enhance MI performance in lower-limb movement imagery.

### 3.3. Performance of UTFB-SSP algorithm

In this section, the classification performance of the proposed UTFB-SSP algorithm was further tested for lower-limb movement imagery decoding. Tables 1, 2 present the classification accuracies of all the algorithms studied for lower limb movement imagery with and without stimulation. The proposed UTFB-SSP achieved mean accuracies of 75.1 and 90.4% for in-house dataset, which are higher than the four competing algorithms, namely, ESI+CNN, GCNs-Net, EEGNet, CSP+SVM, and FBCSP+SVM. Especially, in comparing Tables 1, 2, the accuracies of movement imagery with stimulation are still higher than the accuracies of movement imagery without stimulation. It is thus inferred that multimodal stimulation can help improve the motor imagery classification performance. In addition, to enhance the performance comparison, the classification accuracies of the studied methods are also presented on the public dataset, such as dataset IIa of BCI competition IV. As shown in Table 3, the proposed UTFB-SSP algorithm has higher classification accuracy than the four competing algorithms on the public dataset. The *t*-test analysis of Tables 1–3 are shown in Table 4. Based on the results of the in-house and public datasets, it is concluded that the proposed UTFB-SSP has higher decoding ability than the other state-of-the-art algorithms. This high performance might be attributable in part to the ability of group lasso in the UTFB-SSP method used to automatically select the optimal frequency sub-bands from the upper triangle filter bank.

**Table 1:** Comparison of the accuracies (%) between the proposed algorithm and other methods on the in-house dataset (**without** stimulation).

**Method**	**Mean**	**Subject**									
	**Accuracy**	**A01**	**A02**	**A03**	**A04**	**A05**	**A06**	**A07**	**A08**	**A09**	**A10**
**UTFB-SSD**	75.1	78.3	76.6	73.3	70.0	71.6	81.6	76.6	73.3	76.6	73.3
ESI+CNN	70.9	73.3	71.6	68.3	66.6	68.3	78.3	76.6	68.3	73.3	65.0
GCNs-Net	69.7	71.6	68.3	73.3	68.3	63.3	75.0	71.6	66.6	68.3	71.6
EEGNet	68.4	66.6	70.0	68.3	66.6	65.0	71.6	73.3	68.3	71.6	63.3
CSP+SVM	63.1	61.6	63.3	75.0	61.6	58.3	68.3	60.0	61.6	63.3	58.3
FBCSP+SVM	65.2	68.3	66.6	63.3	63.3	58.3	71.6	61.6	65.0	66.6	68.3

**Table 2:** Comparison of the accuracies (%) between the proposed algorithm and other methods on the in-house dataset (with stimulation).

**Method**	**Mean**	**Subject**									
	**Accuracy**	**A01**	**A02**	**A03**	**A04**	**A05**	**A06**	**A07**	**A08**	**A09**	**A10**
**UTFB-SSD**	90.4	96.6	83.3	98.3	83.3	96.6	86.6	93.3	95.0	95.0	76.6
ESI+CNN	85.1	83.3	78.3	91.6	86.6	93.3	86.6	88.3	86.6	81.6	75.0
GCNs-Net	81.9	76.6	68.3	93.3	78.3	85.0	88.3	86.6	83.3	90.0	70.0
EEGNet	80.4	81.6	75.0	86.6	81.6	88.3	75.0	81.6	78.3	83.3	73.3
CSP+SVM	74.9	68.3	66.6	85.0	71.6	86.6	76.6	78.3	75.0	76.6	65.0
FBCSP+SVM	80.1	70.0	75.0	88.3	78.3	93.3	83.3	76.6	81.6	86.6	68.3

**Table 3:** Comparison of the accuracies (%) between the proposed algorithm and other methods on the BCI competition IV dataset.

**Method**	**Mean**	**Subject**								
	**Accuracy**	**S01**	**S02**	**S03**	**S04**	**S05**	**S06**	**S07**	**S08**	**S09**
**UTFB-SSD**	77.1	81.9	79.1	81.2	90.9	58.3	68.7	93.0	72.2	69.4
ESI+CNN	74.1	79.8	70.1	76.3	86.8	56.2	75.6	83.3	65.9	72.9
GCNs-Net	74.8	74.3	72.9	80.5	81.9	61.1	74.3	88.1	73.6	67.3
EEGNet	73.3	75.6	71.5	76.3	88.8	62.5	70.1	80.5	66.6	68.0
CSP+SVM	71.2	70.1	71.8	74.3	74.3	65.2	72.9	81.9	65.2	65.9
FBCSP+SVM	72.6	76.3	67.3	75.0	79.8	61.8	68.7	82.6	70.1	72.2

**Table 4:** *T*-test results for the proposed method vs. competing method.

**Paired *T*-test**	**Table 1**	**Table 2**	**Table 3**
	***p*-value**	***p*-value**	***p*-value**
UTFB-SSD vs. ESI+CNN	††	*	~
UTFB-SSD vs. GCNs-Net	††	†	~
UTFB-SSD vs. EEGNet	††	††	*
UTFB-SSD vs. CSP+SVM	††	††	*
UTFB-SSD vs. FBCSP+SVM	††	†	*

~ nonsignificant, ^*^p ≤ 0.05,^†^p ≤ 0.005, and^††^p ≤ 0.001.

**Figure 8:**
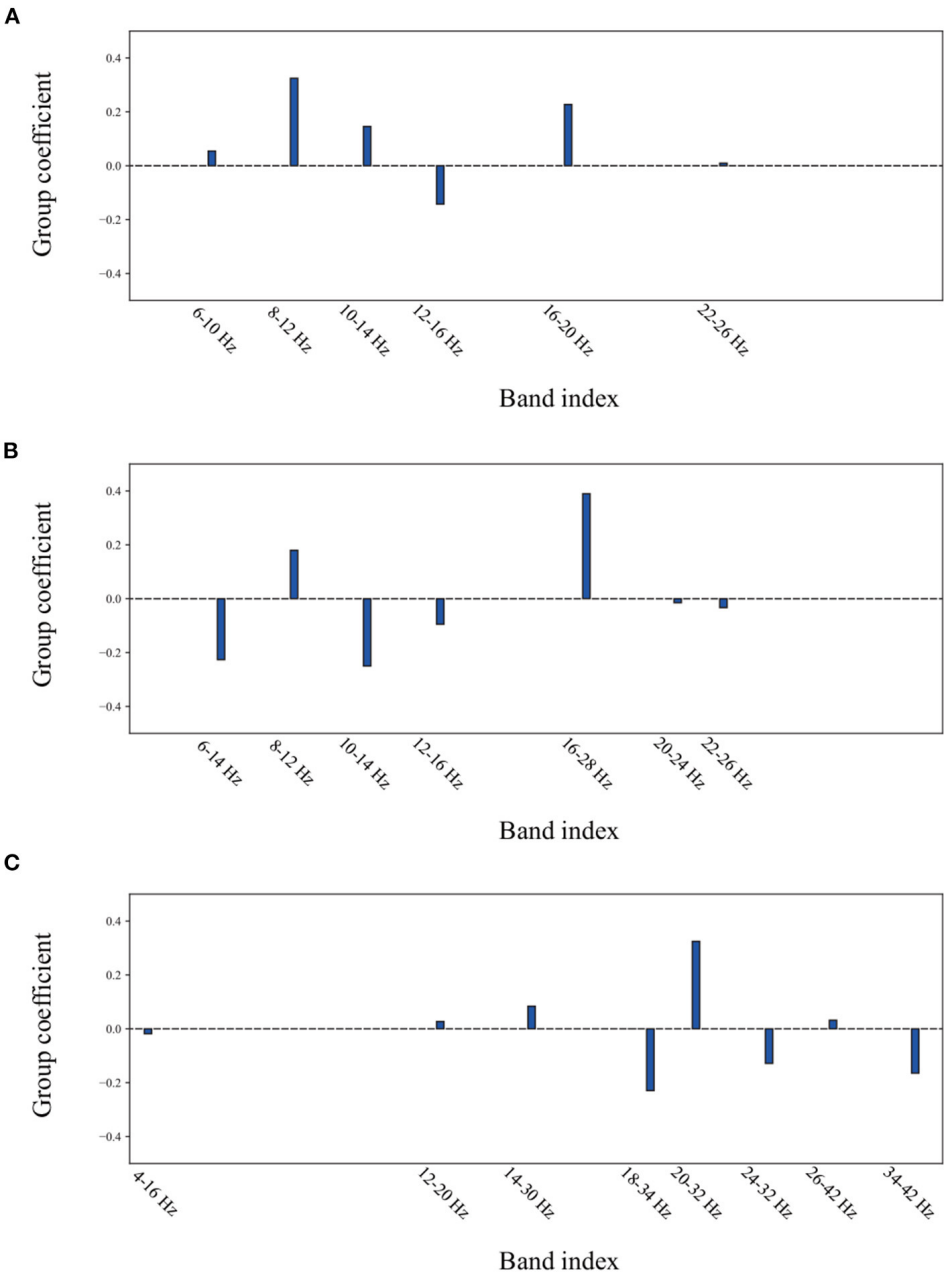
The group coefficient distribution learned by the proposed method from a typical subject. **(A)** the subject A09 from in-house dataset without stimulation. **(B)** the subject A09 from in-house dataset with stimulation. **(C)** the subject S07 from BCI competition dataset IIa.

**Figure 9:**
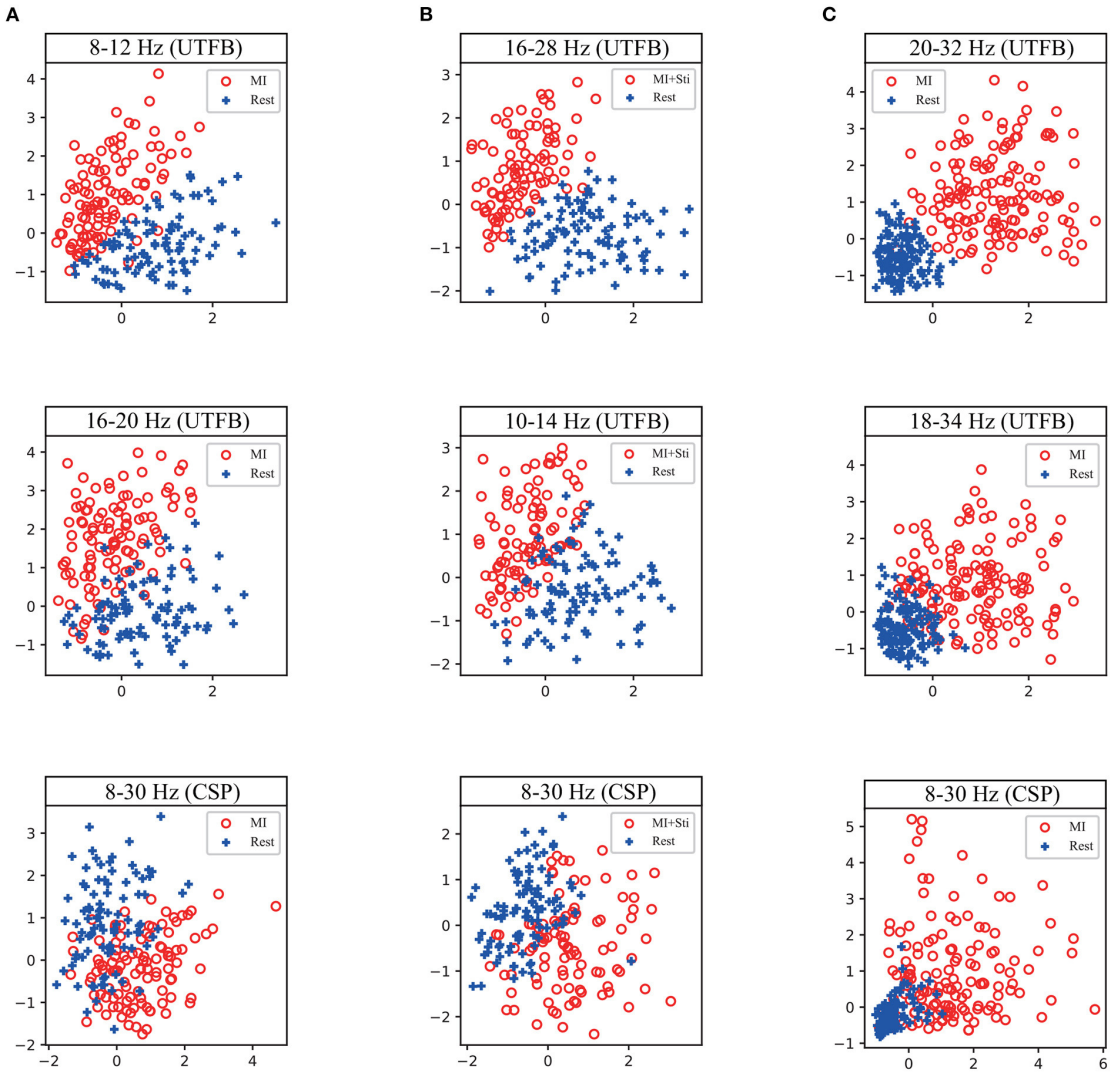
2D feature distribution of each selected sub-band corresponding to the first and second coefficient in Figure 8 from a typical subject. **(A)** the subject A09 from in-house dataset without stimulation. **(B)** the subject A09 from in-house dataset with stimulation. **(C)** the subject S07 from BCI competition dataset IIa.

To reveal the method of optimal frequency sub-band selection using group lasso, an experiment was conducted to show the group coefficient in sparse selection and 2D feature distribution. Figure 8 displays the group coefficient distribution learned by the proposed method from typical subjects A09 and S07. It is observed that the proposed method can precisely select a few key frequency sub-bands from the filter bank. For instance, the sub-bands of 16–28 Hz and 10–24 Hz correspond to subject A09 with stimulation; sub-bands of 8–12 Hz and 16–20 Hz correspond to subject A09 without stimulation; and sub-bands of 8–12 Hz and 12–28 Hz correspond to subject S07 selected by group lasso in the proposed method. To make it more intuitive, the discriminative spatial features learned from the selected sub-bands are presented. Figure 9 shows the 2D feature distribution of each selected sub-band. For a fair comparison, the full-bands of 4–42 Hz were also included in the comparison. It is observed that the feature distribution of the proposed method has a larger between-class scatter and smaller within-class scatter compared to the full band of 4–42 Hz. These results indicate that the features learned by the proposed method have high separability, supporting the possibility of high classification performance, as shown in Tables 1–3.

### 3.4. Relationship of stimulation paradigm and UTFB-SSP

**Figure 10:**
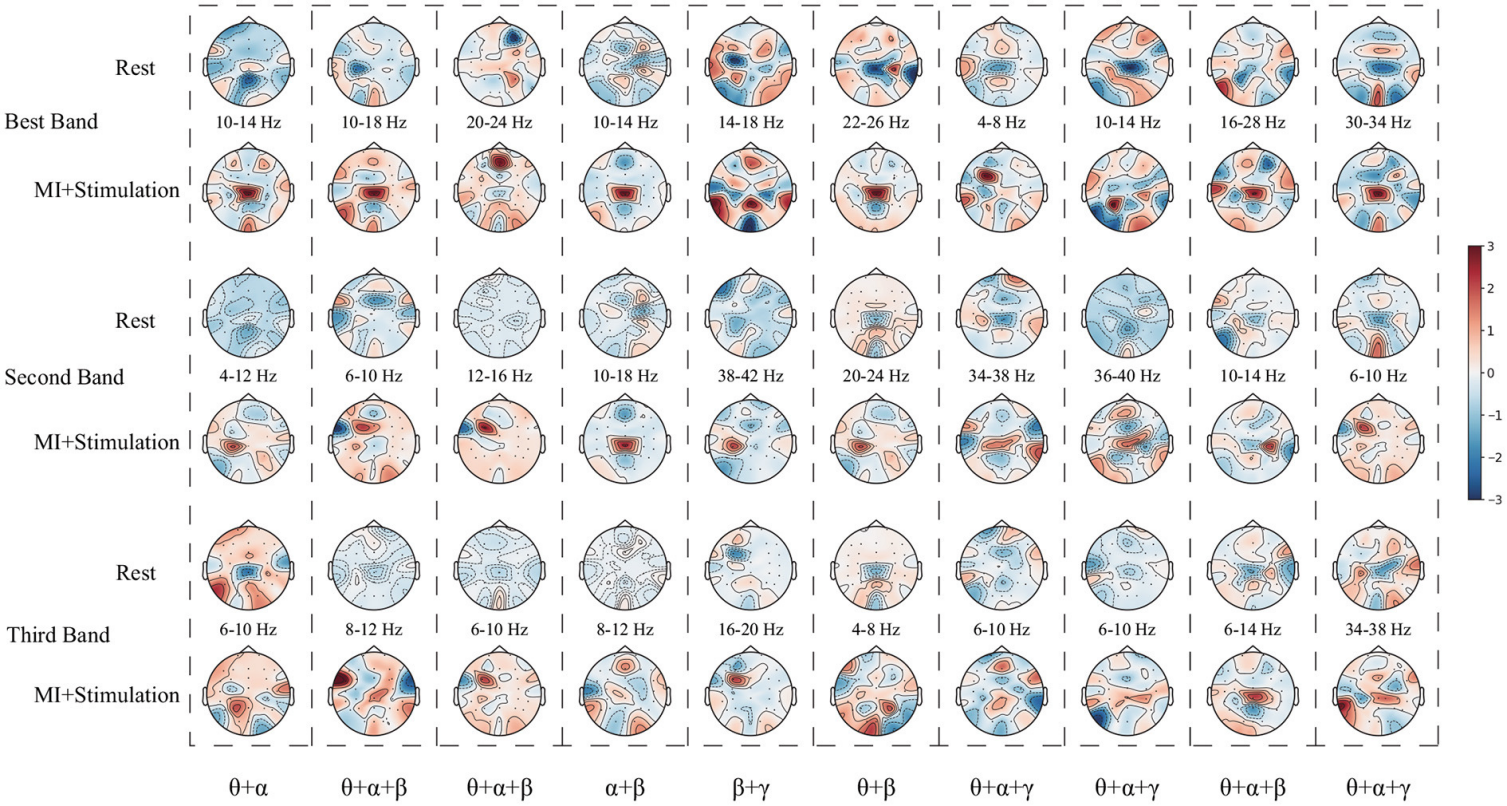
The topographic map learned by the three selected sub-bands corresponding to the top three group coefficients from subjects A01–A10 of the in-house dataset with stimulation.

Finally, the relationship between the stimulation paradigm and UTFB-SSP method is discussed. Figure 10 shows the topographic map learned by three selected sub-bands corresponding to the top three group coefficients from subjects A01–A10 of the in-house dataset with stimulation. The results in Figure 10 indicate that the UTFB-SSP method can automatically select the optimal frequency sub-bands to cover the α, β, and γ rhythms. An interesting result in Figure 10 is that some of the sub-bands selected by the group lasso stretch over the two rhythms. For example, 10–18 Hz of A02 stretches over α (8–13 Hz) and β (14–30 Hz). In the previous results, it was proven that the designed stimulation paradigm can effectively enhance MI performance by inducing α, β, and γ rhythms in lower limb movement imagery tasks. Now, the UTFB-SSP method is recognized for selecting the sub-bands related to the α, β, and γ rhythms. Therefore, the proposed MI-BCI system can provide high performance for motor function rehabilitation after combining the designed stimulation paradigm and the UTFB-SSP method.

## 4. Conclusion

In this study, a multimodal dual-level stimulation paradigm was designed to enhance MI performance and the UTFB-SSP algorithm was proposed to obtain high decoding performance. Compared with other competing methods, the proposed UTFB-SSP method can automatically select the optimal frequency sub-bands using group lasso. The experimental results on the in-house dataset and BCI competition dataset IIa demonstrate the effectiveness of the proposed system. However, rehabilitation experiments were not considered in this study. Future work will involve applying the proposed system to patients with disabilities or stroke patients.

## Data availability statement

The raw data supporting the conclusions of this article will be made available by the authors, without undue reservation.

## Ethics statement

Ethical review and approval was not required for the study on human participants in accordance with the local legislation and institutional requirements. The patients/participants provided their written informed consent to participate in this study.

## Author contributions

XX and YH contributed to conception and design of the study. JX organized the database. FQ performed the statistical analysis. YH wrote the first draft of the manuscript. XX and FQ wrote sections of the manuscript. ZG and ZY revised the manuscript. XX and RT supervised the study. All authors contributed to manuscript revision, read, and approved the submitted version.

## Funding

This work was supported in part by Hainan Province Science and Technology Special Fund under grant ZDYF2022GXJS009, the Young Talents Science and Technology Innovation Project of Hainan Association for Science and Technology under grant QCXM202011, the National Natural Science Foundation of China under grant 61906048, and the Guangdong Basic and Applied Basic Research Foundation under grant 2020A1515010350.

## Conflict of interest

The authors declare that the research was conducted in the absence of any commercial or financial relationships that could be construed as a potential conflict of interest.

## Publisher's note

All claims expressed in this article are solely those of the authors and do not necessarily represent those of their affiliated organizations, or those of the publisher, the editors and the reviewers. Any product that may be evaluated in this article, or claim that may be made by its manufacturer, is not guaranteed or endorsed by the publisher.
